# Supplementary material for: Genome-Wide Identification and Characterization of Tomato Fatty Acid β-Oxidase Family Genes KAT and MFP
Source: Int J Mol Sci. 2024 Feb 14;25(4):2273. doi: 10.3390/ijms25042273 (PMC10889323; doi:10.3390/ijms25042273)
Supplement: Supplementary file 1 [file ijms-25-02273-s001.zip › ijms-2847012-supplementary.pdf]

**Table S1 Primers for qRT-PCR for expression analysis of the *SlKAT* and *SlMFP* family genes in tomato.**

| Gene name | Primer sequence (5ˊ to 3ˊ) | |
|---|---|---|
| *SlKAT1* | F: CGCCTATGGGTGGTTTCCTTG | R: CGCTGAGAACATTTCCAAAGAAGAC |
| *SlKAT2* | F: CCACCATTGTTGATAAGGATGAAGG | R: GAAGCGTTACCAGCCGTCAC |
| *SlKAT3* | F: CAGCAGCAGCAGATACCATCAAG | R: GGCAACGAGGAAAGAGAACCAAG |
| *SlKAT4* | F: AGATACTGTGCCAATCAGAACTGTG | R: CCAATGCCTATGTCGTAGAATCCTG |
| *SlKAT5* | F: GATATTGTTGTTGGCTCGGTGTTG | R: CAGTTCTAATTGGCACGGTTTCAG |
| *SlMFP1* | F: AAGTCAATGCTTGTTCCACTAATGC | R: GCTTGGCTTTGCGTCTTTCATC |
| *SlMFP2* | F: AGGTGGCTTAGAATTGGCATTGG | R: GCCTTGGAAGACGCTGTGTAC |
| *SlMFP3* | F: CACCCGTTAATGCCTTGACTCTC | R: ACCATCAGCACCAGTAAGAACAATG |
| *SlMFP4* | F: GCAGCAGGACAAAGCAAACAAAG | R:GCAGCAGGACAAAGCAAACAAAG |
| *SlActin* | F: AATGAACTTCGTGTGGCTCCAGAG | R: ATGGCAGGGGTGTTGAAGGTTTC |
